# Implementing and Evaluating a Stakeholder-Driven Community Diffusion–Informed Early Childhood Intervention to Prevent Obesity, Cuyahoga County, Ohio, 2018–2020

**DOI:** 10.5888/pcd19.210181

**Published:** 2022-01-20

**Authors:** Larissa Calancie, Deanna Nappi, Julia Appel, Erin Hennessy, Ariella R. Korn, Jodi Mitchell, Alison Patrick, Kelsey Werner, Christina D. Economos

**Affiliations:** 1 Gerald J. and Dorothy R. Friedman School of Nutrition Science and Policy, Tufts University, Boston, Massachusetts; 2 JC Health Strategies, LLC, Twinsburg, Ohio; 3 Cuyahoga County Board of Health, Parma, Ohio; 4 Boston College School of Social Work, Chestnut Hill, Massachusetts

## Abstract

**Purpose and Objectives:**

The purpose of this article is to demonstrate and evaluate aspects of a Stakeholder-Driven Community Diffusion (SDCD)–informed intervention with a group of stakeholders drawn from a large coalition seeking a novel approach for promoting policy, systems, and environmental-level change. The objectives were to implement an SDCD intervention, assess changes in participants’ perspectives, and evaluate where the group’s actions fit within the context of a systems map that the group created during the intervention.

**Intervention Approach:**

An SDCD-informed intervention convened 12 multisector stakeholders from the Early Ages Healthy Stages coalition in Cuyahoga County, Ohio. They participated in group model building activities to promote systems thinking related to childhood obesity prevention, reviewed evidence about topics of interest to the group, and were provided with technical assistance and seed funding to guide the selection and implementation of actions prioritized by the group.

**Evaluation Methods:**

Data were collected via meeting notes and group model building outputs to demonstrate implementation and action prioritization; online surveys and qualitative interviews to measure perspective change among stakeholders; and a follow-up survey to the broader coalition assessing actions coalition members were taking.

**Results:**

An SDCD-informed intervention guided the development of a systems map and the selection of 4 actions: 1) develop a better understanding of the local early childcare environment; 2) assess the effectiveness and impact of Ohio Healthy Programs (OHP); 3) advocate for OHP and improved early childhood education quality; and 4) hold OHP designees accountable to high-quality programming. Data collected from surveys and interviews showed increased awareness of programs, resources, and collaboration opportunities among stakeholders. Follow-up survey results showed ongoing coalition action throughout the systems map.

**Implications for Public Health:**

Using an SDCD-informed intervention among a coalition of community stakeholders provided a unique approach for implementing, assessing, and analyzing collaborative efforts to prevent childhood obesity in Cuyahoga County. Our approach can be applied to help researchers and stakeholders improve efforts to address childhood obesity in their communities.

**Summary**

**What is already known on this topic?**

Obesity is a complex problem with many interconnected drivers and impacts. Combining systems science thinking and policy, systems, and environmental-level change, as implicated in the Stakeholder-Driven Community Diffusion (SDCD) theory, has been successful in addressing obesity.

**What is added by this report?**

This report describes how an SDCD-informed intervention was used to engage a small group of multisector stakeholders drawn from an existing coalition to collectively prioritize obesity prevention action steps and work together to implement those steps. The report also describes use of a systems map to evaluate where action was taking place after the intensive intervention phase concluded.

**What are the implications for public health practice?**

The findings from this report offer an approach for community coalitions interested in using a theory-informed approach for facilitating policy, systems, and environmental-level changes to promote healthy weights among children in their communities.

## Introduction

Many public health challenges, including obesity, are complex in that they are driven by multiple factors that interact over time (1,2). Applying a socioecological perspective, obesity is influenced by individual-level factors (eg, genetics, taste preferences, food preparation skills), social factors (eg, cultural traditions, socioeconomic status), and environmental factors (eg, access to healthy food and safe places to be active) (3). Preventing excess weight gain during childhood is important for reducing obesity rates across the life course (4), and many childhood obesity prevention interventions have targeted single and multiple levels of the socioecological model with varying success (5). Successful obesity prevention interventions often include multiple strategies that target the social and physical environments to influence individual-level behaviors (6). Targeting the policies, systems, and environments (PSEs) that shape healthy eating and physical activity can influence population-level health at a lower cost than individual-level interventions and may address drivers of obesity-related health disparities (7–9). Therefore, federal agencies including the US Department of Agriculture and the Centers for Disease Control and Prevention recommend PSE change for childhood obesity prevention (10–12).

PSE change adoption, implementation, and maintenance requires buy-in from various stakeholders and collaboration across settings and sectors (13). Cross-sector collaborations, including community coalitions, can facilitate PSE change by creating opportunities for individuals and organizations to build trusting relationships, share information, pool resources, and align efforts toward a common goal that is difficult for a single entity to achieve (14). The mechanisms through which cross-sector collaborations create the conditions for PSE change are not well understood, and interventions designed to influence such mechanisms are needed.

Stakeholder-Driven Community Diffusion (SDCD) is a theory that aims to address that gap by proposing a mechanism of how cross-sector collaborations such as community coalitions influence stakeholder members, and in turn, how these members influence the PSEs that shape child health (15,16). SDCD builds on the Community Coalition Action Theory and community-based participatory research by identifying key individual-level factors — stakeholder knowledge and engagement — that may be influenced by coalition participation (14,17,18). SDCD integrates concepts from Diffusion of Innovations Theory and Social Network Theory to explain how changes in knowledge and engagement permeate professional networks (19,20).

Our research team used the SDCD theory to inform an intervention that targets specific constructs and processes. The intervention was pilot tested (21) and was then implemented in Cuyahoga County, Ohio; that implementation is the focus of this study.

## Purpose and Objectives

The purpose of this study was to evaluate aspects of an SDCD-informed intervention with a group of stakeholders drawn from a large, existing community coalition seeking a novel approach for promoting PSE change in their community. The objectives of the study were to assess changes in participants’ perspectives after taking part in the intervention and evaluate where the group’s actions fit within the context of a systems map that the group of convened stakeholders created during the intervention.

### Coalition description

Early Ages Healthy Stages (EAHS) is a coalition led by the Cuyahoga County Board of Health focused on early childhood health and wellness in Cuyahoga County, Ohio. The EAHS coalition grew out of a 2014 task force that was assembled by a group of funders, political leaders, and decision makers to reduce early childhood obesity in Cuyahoga County. EAHS now includes 85 agencies representing sectors influencing early childhood health (eg, health care, home-based and center-based childcare, education providers, social service agencies, community organizations, businesses). The coalition supports programs and initiatives by providing technical assistance and promotes resource and information sharing between member organizations. One initiative is the Ohio Healthy Programs (OHP), a free training and technical assistance program for early childcare and education professionals in Ohio, focused on promoting policies and practices that encourage healthy eating, physical activity, and family engagement strategies to prevent and reduce early childhood obesity (22). OHP includes PSE strategies that support these healthy behaviors and aligns with the Centers for Disease Control and Prevention’s (CDC’s) Spectrum of Opportunities Framework for State-level Obesity Prevention Efforts (23). Research has shown the effectiveness of training programs like OHP on increasing childcare providers’ knowledge and practices (24,25). Given this, EAHS plays a key role in supporting the expansion of OHP through community collaboration and resource sharing, and the expansion of OHP in Cuyahoga County is specified in the EAHS strategic plan.

Despite widespread community support for early childhood health and EAHS’s strategic goal of expanding OHP in Cuyahoga County, the coalition’s work remained siloed within sectors while nontraditional partners such as mental health care providers struggled to see the role that they and their organizations played in early childhood obesity prevention in Cuyahoga County. These barriers inspired EAHS leadership to investigate how a systems approach could unite community efforts, leading to the development of a partnership with ChildObesity180 at Tufts University (https://childobesity180.org/) to implement and evaluate an SDCD-informed intervention in their community.

## Intervention Approach

### Intervention overview

An SDCD-informed intervention was implemented by a team composed of Tufts University researchers, a community-based system dynamics expert at Boston College School of Social Work, a Cuyahoga County Board of Health staff member and EAHS leader, and an external consultant (this group will henceforth be referred to as the “research team”). The group of multisector stakeholders that was convened for the intervention was called the Action Building Committee (henceforth referred to as the “Committee”) and included 12 key stakeholders selected from the EAHS Coalition. EAHS leaders identified 10 Committee members, with input from the research team on sector representation. The 2 remaining positions were chosen by coalition-wide nomination. The Committee represented 8 sectors: nutrition assistance programs, early education, center-based childcare, home-based childcare, public health department, community-based organization, private business, and philanthropy. The Committee received a stipend for participating in approximately 50 hours of intervention and evaluation activities over 10 months.

The SDCD-informed intervention was implemented in 2 phases in Cuyahoga County: 1) an intensive phase in which the research team facilitated Committee meetings and 2) a technical assistance phase in which the research team, which included EAHS leadership, continued to work to advance priorities identified by the Committee. The first step of the intervention was to convene the Committee (Table 1). Next, the Committee engaged in group model building to better understand the systems influencing childhood obesity and facilitate shifts in perspective through exposure to new ideas. Group model building is a participatory approach for engaging stakeholders in building system dynamics models that depict how elements within a system interact to produce patterns of behavior over time (26). Group model building is designed to promote comprehensive understanding of a problem and shared insights among stakeholders, often resulting in increased motivation to implement action steps identified by the group (27). Group model building has been used with community stakeholders globally to create systems models related to childhood obesity and to identify opportunities to reshape those systems through PSE changes that promote healthy child weights (28–31). The SDCD-informed intervention extends existing literature conceptualizing group model building as an intervention that influences participants’ thinking, decision making, and group cohesion (32). During the SDCD-informed intervention, the research team shared evidence (eg, recommendations from consensus reports, findings from peer-reviewed literature) related to the topics that the group prioritized through group model building activities. By using the system insights developed during group model building and the evidence shared by the research team, the group decided what PSE actions they wanted to take to promote healthy weight. The research team provided technical assistance and $20,000 in seed funding to support the group’s actions and to pursue additional funding opportunities to support their work. The research team worked with the group to create a large systems map that has since been used to communicate the complexity and interconnectedness of systems that influence childhood obesity. According to SDCD theory, the intervention activities should spur collaboration and diffusion of knowledge, engagement, and systems insights and facilitate PSE changes aiming to improve child health outcomes (16).

**Table 1:** Timeline of the Implementation and Evaluation of a Stakeholder-Driven Community Diffusion–Informed Intervention to Prevent Early Childhood Obesity, Cuyahoga County, Ohio, 2018–2020

Intervention activities	2018									2019					2020
	Apr	May	Jun	Jul	Aug	Sep	Oct	Nov	Dec	Jan	Feb	Mar	Apr	May	Apr
Committee identification	X														
Committee meetings		X	X	X	X	X	X	X	X	X	X				
Group model building activities		X	X	X	X	X	X	X							
Evidence shares			X	X	X	X	X	X	X	X	X				
Technical assistance and grant-funded action															
Early childhood education scan											X				
Ohio Healthy Programs evaluation												X			
Early Childhood Advocacy Day														X	
Designed Ohio Healthy Programs monitoring tool														X	
Evaluation															
Survey with perspective items						X				X					
Interviews		X								X					
Meeting notes		X	X	X	X	X	X	X	X	X	X				
Group model building outputs		X	X	X	X	X	X	X							
Follow-up survey with entire EAHS coalition^a^															X

Abbreviation: EAHS, Early Ages Healthy Stages.

a EAHS is a coalition of 85 agencies that represent various sectors that influence early childhood health in Cuyahoga County, Ohio.

### Group model building and meeting facilitation

In Cuyahoga County, the intervention included monthly meetings with the Committee that were facilitated by the research team. The first 7 meetings used group model building to gain a deeper understanding of factors driving and impacted by early childhood health in Cuyahoga County. The research team used free group modeling building scripts from Scriptapedia to plan and facilitate group model building activities (33). Scripts provide detailed explanation of inputs needed to conduct group model building activities, how to facilitate the activities, and what outputs the activities should yield (27). The following group model building activities were selected and tailored to the community by the research team: hopes and fears, graphs over time, variable elicitation and connection circles, creating causal loop diagrams from connection circles, initiating and elaborating a causal loop diagram, and action ideas (Table 2). After completing the group model building activities, the Committee developed an action plan and designated existing organizations to operationalize the action items during the final 3 Committee meetings. Insights from the SDCD-informed intervention helped the EAHS coalition develop action strategies to implement the current EAHS strategic plan and to inform future coalition goals and objectives.

**Table 2:** Meeting Themes, Meeting Summaries, and Description of Facilitated Group Model Building^a^ Activities, Early Ages Healthy Stages Action Building Committee,^b^ Cuyahoga County, Ohio, May 2018–February 2019

Meeting theme	Meeting summary and attendance	Description of group model building activity
Project overview and creating a shared vision	• Committee and research team introductions • Project overview (introduction to group model building and systems dynamics) • Hopes and fears (group model building) • Shared vision group discussion • Attendance: 11 of 12	**Hopes and fears**: Prompted with the question, “What are your hopes and fears for our work together over the next 10 months?” Early Ages Healthy Stages Committee members shared their personal hopes and fears for the project and, with the help of the facilitators, organized responses into themes.
Identifying trends and systems	• Evidence share by research team: Connecting early childhood education and health • Hopes and fears recap • Graphs over time (group model building) • Group discussion • Attendance: 9 of 12	**Graphs over time**: Given the prompt, “What impacts, or is impacted by, the work of the Early Ages Healthy Stages Coalition?” members identified factors that fit the description then created and shared line graphs showing how they perceived these factors to have changed in recent decades and the potential future trajectories that they hoped and feared might unfold.
Identifying and connecting system variables	• Graphs over time recap • Variable elicitation (group model building) • Connection circles (group model building) • Group share and discussion • Attendance: 9 of 12	**Variable elicitation**: Guided by the questions, “What are key things that affect the functioning of the Early Ages Healthy Stages Coalition, or the impact that the Coalition has in the community?” members wrote variables that came to mind. **Connection circles**: Members worked in groups to draw connections between variables around a circle, using arrows to begin seeing how variables can be connected.
Reflecting on the past and sketching a roadmap	• Evidence share by research team: The importance of early learning • Reflection on prior activities (hopes and fears, graphs over time, variable elicitation, connection circles) • Connection circles (focused on connection between coalition’s functioning and impact) • Group share and discussion about defining success moving forward • Attendance: 9 of 12	[See description of Hopes and fears, Graphs over time, Variable elicitation, and Connection circles]
Visualizing systems connections and structures	• Evidence share by research team: Promoting community health improvement through more equitable food systems • Introduction to causal loop diagrams: Purpose and use in Committee • Introduction to causal loop diagrams: Technical aspects and mechanics of drawing (group model building) • Small group drawing of causal loop diagrams • Group share • Causal loop diagram combination by facilitation team (during group lunch break) • Reaction and refinement of combined causal loop diagram as whole group • Attendance: 11 of 12	**Causal loop diagrams**: Committee members learned how to read and create causal loop diagrams. A causal loop diagram was then developed by the entire group to visualize connections between factors identified in previous group model building activities and identify system structures, such as feedback loops, that drive trends over time. Creating a causal loop diagram helps groups develop shared language and begin to understand the dynamics of a complex problem.
Causal Loop Diagram elaboration and use for action planning as systems map	• Evidence share by research team: Assessment of child health and health care in Ohio • Research team presented integrated causal loop diagram, review of causal loop diagram, progression, summary of key feedback loops (group model building) • Small group discussion: Is there anything missing or that should be changed? • Group share and discussion • Attendance: 10 of 12	**Causal loop diagram elaboration**: The causal loop diagram was updated between meetings by the research team and then presented back to the group. When presented back, the facilitator explained each loop and reflected on key insights before asking the group for feedback on what is missing. Refining and elaborating the causal loop diagram as a group ensures that all connections are included and that all members feel represented. This causal loop diagram was styled into a systems map to be used primarily as a communication tool moving forward.
Identifying opportunities for systems change	• Evidence share by research team: Overweight/obesity and blood pressure in Cuyahoga County • Review of refined systems map and feedback loop connections • Reflection on the importance of systems change • Individual work to generate action ideas (group model building) • Group share and impact-feasibility grid • Group discussion • Attendance: 7 of 12	**Action ideas**: Action ideas that targeted specific areas of the systems map were then conceptualized using an impact-feasibility grid, a tool to guide members in formulating actionable solutions, and creating a shared understanding of potential interventions within the system.
Prioritizing activities for action ideas	• Evidence share by research team: Early Ages Healthy Stages engagement in the community • Presentation of top 11 survey results (Committee members voted on top ideas to prioritize from impact-feasibility grid) • Presentation of evidence around top strategies, developed by research team • Group discussion of each idea and what needs to happen to move forward • Attendance: 7 of 12	NA
Action planning and catalyzing future work	• Evidence share by research team: The intersection between health and education in very young children • Action planning continued: more structured discussion around top 4 action items • Introductory discussion to sustainability • Attendance: 9 of 12	NA
Preparing for sustainability of work going forward	• Evidence share by research team: Roundtable discussion on obesity solutions with local early childhood education leaders • Discussion of action strategies moving forward using the systems map • Committee Culmination/Kickoff event with community leaders to showcase work: panel discussion on creating healthier early childhood environments through community • Group discussion on sustainability of work moving forward • Hopes and fears (for future) • Attendance: 3 of 12	NA

Abbreviation: NA, not applicable.

a Group Model Building is a participatory approach for engaging stakeholders in building system dynamics models that depict how elements within a system interact to produce patterns of behavior over time.

b Early Ages Healthy Stages is a coalition of 85 agencies that represent various sectors that influence early childhood health in Cuyahoga County, Ohio. Attendance numbers reflect people who attended the regularly scheduled meetings, not those who attended the make-up meetings.

The research team planned the monthly meetings. Each meeting followed a general structure: introduction, brief evidence shares, group model building activity, group discussion, and reflection (Table 2). However, the process was flexible in that meeting plans could be adapted on the basis of Committee feedback. The research team facilitated monthly make-up meetings (conducted in person or over the telephone, depending on participant availability), as close to the scheduled meeting date as possible, to ensure that all individuals had a chance to share their ideas and perspectives. Make-up meeting participants also received a summary of the discussions and takeaways from the scheduled meeting.

Starting with the second meeting, the research team shared evidence from peer-reviewed and gray literature related to each meeting theme to increase Committee members’ understanding of topics related to early childhood health. In response to Committee members’ expressed interests, evidence share topics included the connection between early childhood education and health, promoting community health through equitable food systems, assessment of child health and health care in Ohio, and a roundtable discussion on obesity solutions with local leaders.

## Evaluation Methods

Data collection included meeting notes and group model building outputs to demonstrate implementation, online surveys and interviews to assess Committee member perspective shifts, and a follow-up survey to identify actions taken by the EAHS following the SDCD-informed intervention with the Committee (Table 1).

### Implementing an SDCD-informed intervention

The research team recorded meeting notes at each Committee meeting to document meeting facilitation, activities, and actions that the group prioritized. Additionally, Committee members developed tangible outputs during group model building activities, including graphs and system maps that depict key concepts and relationships discussed by the group (34). The research team photographed group model building outputs at the conclusion of meetings. Throughout the study period, Committee members revisited group model building outputs to reinforce their understanding of the underlying relationships impacting early childhood obesity and to discuss ways in which to intervene (34).

### Perspective shifts

Committee members were surveyed about shifts in their perspective related to early childhood obesity prevention in Cuyahoga County. Participants were asked via multiple-choice questions whether they had experienced a perspective change related to early childhood obesity prevention and whether a person in the Committee, participation in the Committee, or both influenced the change. Questions about perspective shifts were embedded in a longer survey that took approximately 20 minutes to complete. The survey was written in English and disseminated using Qualtrics, an online survey platform, and was administered during months 5 and 9 of Committee meetings. Only data from month 9 were used in this study to assess perspective shifts at the final stage of Committee meetings. Survey questions about perspectives shifts were tallied and frequencies were reported.

One member of the research team (J.A.) conducted interviews with Committee members at baseline and at the conclusion of the study. That member of the research team also assisted with Committee meeting facilitation. The same interview questions were asked at both points. An SDCD-informed interview guide was designed to capture perspective changes related to early childhood health and early childhood obesity prevention between baseline and the end of Committee meetings (15). Questions also asked about Committee members’ thoughts on the SDCD process and how their participation in the Committee influenced their perception of early childhood health and obesity prevention. Interviews were transcribed and responses were summarized into themes related to perspective shifts and reflections on the SDCD-informed intervention by one of the co-authors (D.N.) who was not involved in Committee meetings.

### Follow-up action survey

Fourteen months after the conclusion of Committee meetings, the research team distributed another online survey to all members of the EAHS coalition (n = 387) to understand the actions that had been taken related to early childhood health in Cuyahoga County. This survey was different than the one used to assess shifts in perspectives. The survey was sent 14 months after the Committee meetings ended to capture actions that may have been inspired by the intervention but that take time to initiate. Using the systems map developed by the Committee (Figure), participants selected up to 5 variables on the map to indicate where participants had taken action related to early childhood obesity prevention. The results of the survey allowed the research team and EAHS coalition leaders to assess where within the system there was the most and least activity. The design of this systems map–based action survey was informed by a similar evaluation activity developed for the WHO STOPS project (35).

**Figure:**
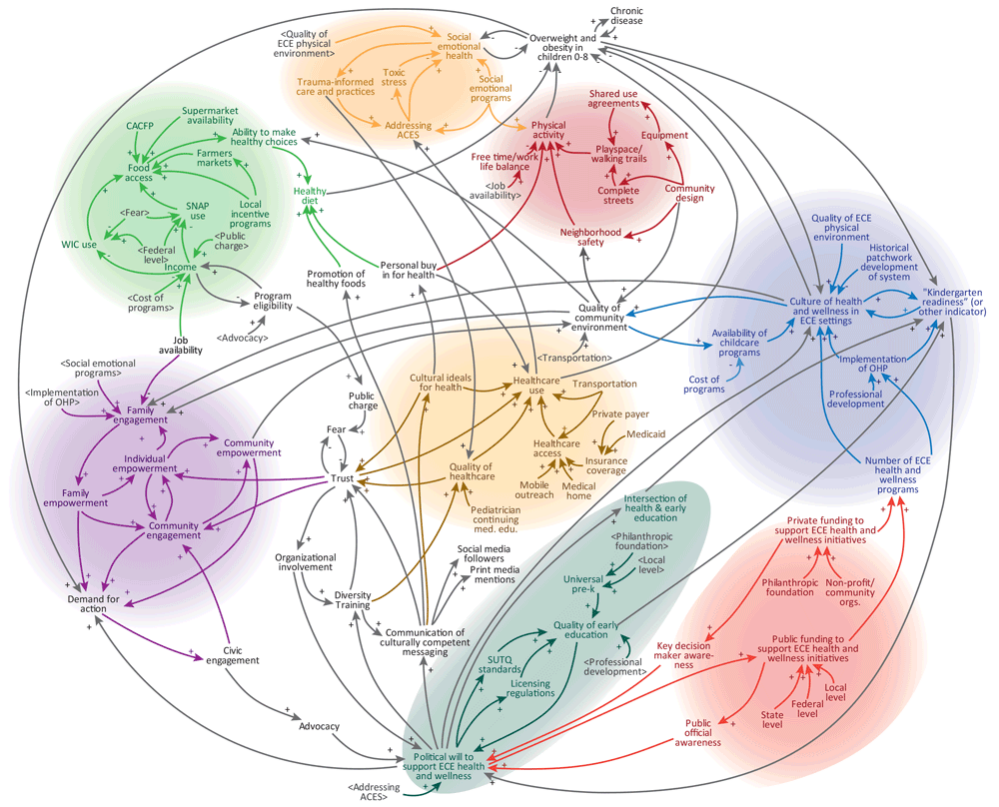
Systems map developed and refined by the Early Ages Healthy Stages Action Building Committee, 2019. Arrows indicate a connection between variables; plus signs indicate a connection in which change in one direction (eg, an increase or decrease) in the first variable results in a change in the same direction in the connected variable; minus signs indicate a connection in which a change in one direction in the first variable results in a change in the opposite direction in the connected variable. Abbreviations: ACEs, adverse childhood experiences; CACFP, Child and Adult Care Food Program; ECE, early childhood education; OHP, Ohio Healthy Programs; SNAP, Supplemental Nutrition Assistance Program; WIC, Special Supplemental Nutrition Program for Women, Infants, and Children.

## Results

### Implementing an SDCD-informed intervention

#### Meetings and group model building activity outputs

Throughout the study, an average of 90.7% of the 12 Committee members attended either originally scheduled (range, 7–12 attendees) or make-up meetings (range, 1–4 attendees), except for the final meeting. Only 3 Committee members attended the final meeting due to poor weather and the meeting being scheduled on a holiday. Committee members who did not attend received an email that provided information from the meeting. Committee members produced outputs throughout the series of group model building activities, including graphs depicting obesity-related variables changing over time and multiple versions of a causal loop diagram (Table 2). Over several sessions, the diagrams maintained their connection to participants’ understanding of the system and relationships between variables but lost some connection to the principles of system dynamics (34). The diagrams evolved into systems maps, rather than a causal loop diagram representing a specific dynamic hypothesis. Therefore, the diagrams were merged into 1 systems map and styled to highlight different thematic areas in the system (Figure).

The final systems map developed by the Committee included 81 variables, organized into 8 main themes: healthy diet, engagement, political will, health care, physical activity, social emotional health, funding, and health and wellness in early childhood education settings (Figure). The systems map was primarily used to inform conversations around action prioritization and implementation. EAHS leadership continues to use this map as a tool for developing partnerships, unifying cross-sector efforts, and communicating to stakeholders how specific actions can influence the broader goal of increasing early childhood wellness in Cuyahoga County.

An impact feasibility grid was the last group model building output from the Committee. Committee members brainstormed intervention ideas or actions they could take to improve the system depicted in their systems map and ranked each idea based on its potential impact and feasibility. Next, the group voted on the items, reviewed relevant scientific evidence provided by the research team, and ultimately identified 4 actions.

#### Prioritized actions

After participating in group model building activities and reviewing evidence, the Committee prioritized the following actions to promote PSE change in early childcare settings: 1) develop a better understanding of the early childhood education system in Cuyahoga County, 2) assess the effectiveness and impact of OHP, 3) advocate for OHP and improved early childhood education quality, and 4) hold OHP designees accountable to meeting their objectives. Each of these actions reinforced an overarching goal of strengthening OHP, a goal chosen for its broad impact potential as seen in the systems map and impact feasibility grid, as well as its alignment with the EAHS strategic plan.

#### Taking action

The Committee and research team worked together to advance the 4 prioritized actions (Table 3). To develop a better understanding of the early childhood education system in Cuyahoga County, the research team member from the Cuyahoga County Board of Health (the EAHS Coalition leader) conducted a scan of existing efforts and initiatives in Cuyahoga County. The goal of the scan was to understand where opportunities existed to work with ongoing initiatives and where there was a need to advance advocacy efforts and expand OHP into more early childhood education programs. To assess the effectiveness and impact of OHP, the Committee worked with an expert at Kent State University College of Public Health to design and conduct an evaluation of the impact of OHP on early childhood environments by using secondary data collected at OHP sites. This evaluation led to the development of a white paper targeting local and state funders and key decision makers, with the aim of advocating for funding and for the integration of OHP into the Ohio early childhood education quality rating system and/or licensing requirements (36). To develop the positions of EAHS members as child health champions and prepare them to advocate for OHP, one of the research team members hosted an advocacy training for EAHS members before an early childhood advocacy day in May 2019 at the state capitol. Twelve coalition members attended the training, and 4 EAHS representatives attended the statewide advocacy day. Finally, to hold OHP designees accountable, a monitoring tool to assess fidelity to the OHP designation requirements was developed in collaboration with an OHP coordination and program manager. OHP grant coordinators at the Cuyahoga County Board of Health will use the observation tool to make recommendations to sites.

**Table 3:** Prioritized Actions and Outcomes to Reduce Prevalence of Childhood Obesity, Developed Through the Group Model Building Process, Action Building Committee of the Early Ages Healthy Stages Coalition, Cuyahoga County, Ohio, 2019–2020

Prioritized actions	Implementation activities	Implementation outcomes
Understand early childhood care system in Cuyahoga County	Conducted a scan of existing efforts and initiatives in Cuyahoga County	• Scan survey developed and fielded in spring 2019 • Results of scan presented to Early Ages Healthy Stages coalition subgroup to assist with development of strategic plan in December 2019
Assess the effectiveness and impact of OHP in Cuyahoga County	Designed and conducted an evaluation of OHP	• Evaluation conducted and results presented to Cuyahoga County Board of Health in February 2020 • Manuscript submitted for publication July 2020 • White paper developed to advocate for funding for OHP, and for the integration of OHP into the Ohio early childhood care quality rating system and/or licensing requirements in June 2020
Advocate for OHP and improved early childhood care quality in Cuyahoga County	Developed communication materials and hosted a training to advocate for enhanced integration of early childhood health and education at a state level	• 12 Coalition members attended the training held in March 2019 • 4 Coalition members attended a statewide advocacy day held in May 2019
Maintain accountability of OHP designees in Cuyahoga County	Worked with OHP coordinator and program manager to develop monitoring tool for OHP-designated sites	• Monitoring tool designed summer and fall 2019, with planned implementation of tool in early 2020; implementation delayed due to COVID-19

Abbreviation: OHP, Ohio Healthy Programs.

### Perspective shifts

Survey responses by Committee members showed perspective changes after engaging in group model building activities. After 9 months, 9 Committee members (75%) noted a change in their perspective on early childhood obesity prevention, specifically, an increase in awareness of programs, resources, and collaboration opportunities in the early childhood education setting. Five members indicated that another member of the Committee influenced the change. The 9 members who reported a perspective change all reported that their involvement in the Committee influenced the change, referring to the following specific aspects of their participation: exposure to diverse perspectives, participating in committee meeting activities, working with committee members, exposure to diverse roles, and scientific evidence presented to the Committee.

Similar perspective shifts were reported in qualitative interviews of Committee members that were conducted at baseline and at the conclusion of Committee meetings, with all 12 members of the Committee completing both interviews. The interviews highlighted an appreciation for the Committee experience and new collaboration opportunities within the group (“I enjoyed strengthening the relationships with the other persons that were participating . . . I definitely have a newfound appreciation and feel like I know more now than I did a year ago”). These interviews also demonstrated increased knowledge of resources and of county-wide childhood obesity prevention efforts that are under way (“It’s been enlightening, like I’ve learned a lot about what’s going on in Cuyahoga County and all of the players”), as well as a recognition of systems influencing childhood health in the county (“I really valued . . . understanding at a deeper level what some of the work looks like from the systems perspective, the systems that were represented in the room”).

### Follow-up survey and future coalition work

Sixty-three (16%) of 387 EAHS coalition members completed the follow-up survey 14 months after the conclusion of Committee meetings. Of respondents, 30% identified their primary sector as center-based childcare; 19%, early education; 15%, community-based organization; 11%, home-based childcare; and the remaining 25% of respondents representing health care, Cuyahoga County Board of Health, philanthropic organizations, parents, university, and other sectors. The survey asked participants to select variables they were working on within the systems map generated during group model building activities. Of the 81 variables, the most frequently selected were physical activity (n = 19 selections), family engagement (n = 18), food access (n = 15), social emotional health (n = 15), Child and Adult Care Food Program (n = 12), community engagement (n = 8), Step Up To Quality standards (n = 8), and trauma-informed care (n = 8). Although not conclusive given the low response rate, these variables show ongoing work in the engagement, healthy diet, social-emotional health, and physical activity subsystems of the systems map, while also indicating a potential lack of activity in the political will, funding, health care, and health and wellness in early childhood education settings subsystems. The results of this follow-up survey suggest that sustained efforts are needed in early childhood health, building on the actions prioritized by the Committee. Results of this survey provide EAHS with a rough estimate of where work is ongoing within the system and where the coalition could focus their efforts to reinforce existing efforts or to fill in gaps.

## Implications for Public Health

This study demonstrates how the SDCD theory can inform PSE-level initiatives and sustained community-led action. The study engaged 12 stakeholders from the EAHS coalition in Cuyahoga County to develop a holistic view of the system influencing early childhood obesity in their county and use those insights to generate and implement action in their community. The research team shared information throughout the intervention to encourage the group to select evidence-based actions. The actions focused on the early childcare system in Cuyahoga County and strategies for expanding OHP, which includes PSE approaches to support healthy eating and physical activity among young children. Stakeholders reported that the SDCD theory–informed intervention influenced their knowledge of the problem of early childhood obesity and their awareness of resources and collaboration opportunities to address the problem. Additionally, the follow-up action survey provides a unique approach for assessing ongoing work in a community. The systems map developed by the Committee allows EAHS leadership to better understand what is driving and what is affected by the work of the coalition and create or adapt coalition priorities as necessary.

Our approach in Cuyahoga County responds to the call for researchers and practitioners to use systems thinking and community engagement to promote public health (13,37). Improving complex, adaptive systems (eg, health and social systems) requires collaboration across disciplines, sectors, and organizations (38). Systems science provides approaches and methods for examining interactions between variables over time that shape population health outcomes (39). Systems thinking tools can help groups with different expertise and perspectives build shared understanding and support learning as groups work to address complex public health issues (38). Systems thinking is highlighted as a critical capability to equip public health practitioners to effectively respond to forces rapidly reshaping the field, including climate change, demographic shifts, and social media and informatics (37). Developing effective interventions for population change requires collaboration between sector leaders as well as broad community buy-in. Integrating community-engaged research and systems science by taking a community-based system dynamics approach to build community capabilities in system dynamics offers new methods to study the systems that generate and perpetuate serious public health challenges and strengthens the translation of “knowledge to action” (40).

Our approach is generalizable in many ways. Community coalitions exist across the US, and decades of research indicate that coalitions may be amenable to working with scientists to try new strategies and engage in data collection activities. Conducting group model building activities that yield meaningful systems insights that can be shared within a group takes skill and training, which can be a limiting factor when using group model building to implement an SDCD-informed intervention as described here. Increasing training opportunities for group model building and community-based system dynamics could increase the pool of graduates and professionals who could implement interventions that use those methods. The main costs of implementing this intervention are salaries for those implementing and evaluating the intervention, stipends for intervention participants, and seed funding to initiate community-based actions. Grant funding was secured from a foundation to cover the costs of implementing the intervention described in this study. Finally, the SDCD theory and SDCD-informed intervention could be applied to a variety of public health concerns beyond childhood health, because the theory describes processes and mechanisms not specific to a single public health concern.

This study also has limitations. We cannot isolate the effect of our SDCD process from the effects of general facilitation with a group because we had no control group. Future studies should include a comparison group. Building on systems thinking and modeling capabilities developed within this group, future work using group model building as a process for engaging stakeholders in Cuyahoga County could work toward more rigorous causal loop diagrams or formal system dynamics models with simulation to enable deeper system insights (40). Further, this should include taking a community-based system dynamics approach to engaging stakeholders to enable an explicit emphasis on developing community capabilities to ensure community ownership over system insights (40). The follow-up survey asked participants to click on variables in the systems map where they took or are taking action. There is an opportunity to test the reliability and validity of a similar survey with a more parsimonious causal loop diagram, and to program it such that participants can also select connections between variables and feedback loops to indicate they are working on the relationships between variables that drive system behavior. According to the Meadows Leverage Points framework, influencing connections and feedback mechanisms within a system is likely to create more change in system behavior over time than focusing on individual variables (41). An opportunity exists to administer pre–post surveys to assess change in systems actions. However, doing so would require a baseline systems map, which was not available in this study because the systems map was created as part of the intervention. Additionally, the low response rate of the follow-up survey limits our ability to draw conclusions about actions underway within the systems map after the Committee finished meeting. The low response rate may have been due to the survey timing (ie, COVID-19 was surging in the US), who sent the survey (a member of the research team whose name may have been unfamiliar to survey recipients), and time that the survey was open (3 weeks). Although survey results indicate a lack of activity in political will, funding, health care, and health and wellness in early childhood education settings subsystems, this may reflect who responded to the survey (ie, mostly early childcare professionals). Increasing response rate and sector representation would provide a more accurate assessment of coalition actions.

To conclude, an SDCD-informed intervention offered the EAHS coalition a new approach for member engagement, leading to a large systems map of factors driving childhood obesity and health in Cuyahoga County. This galvanized community-level action intended to improve the system influencing early childhood health in the community. The coalition continues to use the systems map to communicate the interconnectedness of childhood obesity–related factors across sectors. The coalition also uses the systems map to plan and evaluate their work toward their vision.
